# Computational Studies of Glutamate Transporters

**DOI:** 10.3390/biom5043067

**Published:** 2015-11-11

**Authors:** Jeffry Setiadi, Germano Heinzelmann, Serdar Kuyucak

**Affiliations:** 1 School of Physics, University of Sydney, New South Wales, Sydney 2006, Australia; E-Mail: setiadi@physics.usyd.edu.au; 2 Departamento de Fisica, Universidade Federal de Santa Catarina, Florianopolis 88040-900, Santa Catarina, Brazil; E-Mail: germanohei@gmail.com

**Keywords:** glutamate, transporters, computational, molecular dynamics

## Abstract

Glutamate is the major excitatory neurotransmitter in the human brain whose binding to receptors on neurons excites them while excess glutamate are removed from synapses via transporter proteins. Determination of the crystal structures of bacterial aspartate transporters has paved the way for computational investigation of their function and dynamics at the molecular level. Here, we review molecular dynamics and free energy calculation methods used in these computational studies and discuss the recent applications to glutamate transporters. The focus of the review is on the insights gained on the transport mechanism through computational methods, which otherwise is not directly accessible by experimental probes. Recent efforts to model the mammalian glutamate and other amino acid transporters, whose crystal structures have not been solved yet, are included in the review.

## 1. Introduction

Neurons in the central nervous system communicate using organic molecules called neurotransmitters. When an action potential arrives at the synapse of a neuron, it opens the calcium channels. The increased concentration of Ca${}^{2+}$ ions triggers release of neurotransmitters to the synaptic cleft between two neurons. The neurotransmitters diffuse through the cleft and bind to the receptors at the neighboring neuron, some of which are ligand-gated ion channels. Binding of an excitatory neurotransmitter such as glutamate to a receptor opens a cation channel, which depolarizes the membrane potential and increases the probability of generating an action potential [1]. Conversely, binding of an inhibitory neurotransmitter such as GABA or glycine to a receptor, opens a potassium or chloride channel, which hyperpolarizes the membrane potential and thereby suppresses formation of an action potential.

Glutamate is the major excitatory neurotransmitter in the mammalian central nervous system [2]. It thus plays a pivotal role in brain function, and disruption of the processes involving glutamate results in a number of neurological disorders. Glutamate molecules are loaded in synaptic vesicles by vesicular glutamate transporters and released to the synaptic cleft through the action of SNARE proteins [3]. The extracellular concentration of glutamate is in the nanomolar range while, in the cytoplasm, it is in the milimolar range [4]. Excess glutamate in the synaptic cleft results in over-activation of receptors—called excitotoxicity—which leads to neuronal damage and eventual cell death. Such effects have been associated with Alzheimer’s disease [5], amyotrophic lateral sclerosis [6], ischaemia [7] and epilepsy [8]. Thus rapid removal of excess glutamate from the synaptic cleft is essential for normal functioning of neurons.

**Figure 1:**
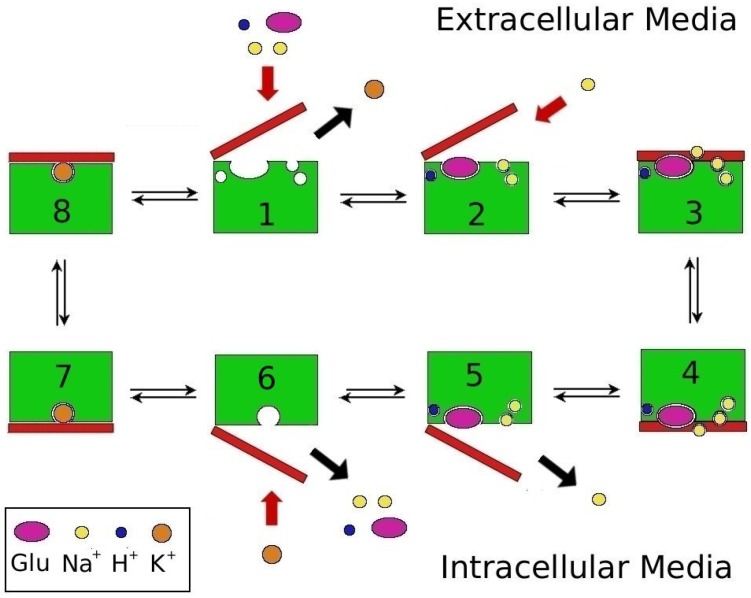
Mechanism of coupled-glutamate transport in EAATs. Step 2 shows the binding of the Na2 ion, which occurs after the binding of the substrate and the closure of the HP2 gate. Step 5 shows the opposite happening in the inward-facing state. Steps 3 and 4 correspond to the translocation of the transport domain across the membrane with 3 Na${}^{+}$, H${}^{+}$, and Glu bound to EAAT, while steps 7 and 8 depicts the same with only K${}^{+}$ bound.

The concentration of glutamate in the synaptic cleft is maintained by specific transport proteins called excitatory amino acid transporters (EAATs) [2]. A cartoon representation of the transport mechanism is shown in Figure 1, which involves two half-cycles. In the first part, three Na${}^{+}$, one H${}^{+}$, and Glu are bound to the outward-facing state and translocated across the membrane. In the second part, this cargo is released to the cytoplasm, followed by binding of a K${}^{+}$ ion which is counter-transported to the outward-facing state and released to the plasma, completing the cycle [9,10,11].

EAATs are part of the solute carrier family 1A, which includes neutral amino acid transporters called Alanine-Serine-Cysteine transporters (ASCTs) and other prokaryotic transporters [12]. There are five known subtypes of EAATs (EAAT1-5) and two ASCTs (ASCT1-2) [13,14,15]. Both EAATs and ASCTs couple the substrate transport to the concentration gradient of Na${}^{+}$ ions and hence belong to the class of secondary active transporters. Within the EAAT family, the subtypes share 50%–60% homology and are known to have the same transport mechanism. In comparison, ASCTs share 30%–40% homology to EAATs and employ a simpler transport mechanism. The transport is independent of H${}^{+}$ and K${}^{+}$ ions, which is similar to the aspartate transporter Glt${}_{\mathrm{Ph}}$, but the number of Na${}^{+}$ ions required for transport has not been established yet.

A major breakthrough in the study of glutamate transporters was the solution of the crystal structure of the prokaryotic homolog Glt${}_{\mathrm{Ph}}$ from *Pyrococcus horikoshii* in the outward-facing conformation [16]. Since then, successive iterations of the crystal structure of Glt${}_{\mathrm{Ph}}$ have been resolved, including the binding sites of the substrate and two sodium ions [17], the inward-facing [18] and intermediate [19] conformations. Following the argument of reductionism, Glt${}_{\mathrm{Ph}}$ provides a relatively simpler model for EAATs and ASCTs. Glt${}_{\mathrm{Ph}}$ shares 36% amino acid sequence identity with EAATs [16] and 23% with ASCTs [14]. Although the sequence identity is low for the overall protein, the sequence identity in the binding pocket is as high as 60%. Glt${}_{\mathrm{Ph}}$ differs from EAATs in its transport cycle in that it does not require the co-transport of H${}^{+}$ or the counter-transport of K${}^{+}$, and is selective for aspartate [20]. For a thorough discussion of the experimental work on Glt${}_{\mathrm{Ph}}$ and its relevance to EAATs, we refer to a recent review article [21].

Determination of the crystal structure of Glt${}_{\mathrm{Ph}}$ has opened the way for computational investigation of glutamate transporters. Many atomistic molecular dynamics (MD) and coarse-grained simulations of Glt${}_{\mathrm{Ph}}$ have been performed to obtain further insights on the mechanism and energetics of aspartate/glutamate transport. In this review, we focus on the computational work involving the crystal structure of Glt${}_{\mathrm{Ph}}$. In particular, we discuss how computational methods are used to model and probe the transport mechanism and energetics that are otherwise difficult to access using experimental techniques. In addition to the work on Glt${}_{\mathrm{Ph}}$, recent computational efforts to construct homology models of EAATs and ASCTs based on the Glt${}_{\mathrm{Ph}}$ structure are discussed.

## 2. Computational Methods

### 2.1. Molecular Dynamics

MD is the *de facto* method for computational studies of biomolecules at atomic resolution. MD simulations can describe the motion of a many-body system accurately enough to compare with experimental results. In MD simulations of a system of *N* interacting particles, one integrates the classical equations of motion numerically to find the state of the system at later times. The accuracy of the method depends mainly on the potential functions used to describe the interactions between particles, which are collectively called a force field. The non-bonded interactions in a force field consist of the Coulomb and 12-6 Lennard-Jones potentials, the latter describing the dispersion and short-range repulsion between atoms. Covalent bonds in a molecule are represented by simple harmonic potentials for bond lengths and angles while more complex forms are required for the dihedral interactions involving four neighboring atoms [22]. Popular force fields used in MD simulations of biomolecules include AMBER, CHARMM, GROMOS and OPLS. There are also a number of popular MD programs that are used with these force fields, e.g., AMBER, CHARMM, GROMACS, and NAMD. We note that the polarization interaction has not been incorporated in the current versions of these force fields yet. Because the force fields are optimized for proteins in bulk solution, one has to be careful with simulations involving interfaces, narrow channels and multi-valent ions, where polarization effects may not be accounted properly.

Many processes in molecular biology occur on a time scale that is much longer than can be reached in a typical MD simulation. Thus, in order to fully describe a biomolecular process, it is essential to accelerate MD simulations using enhanced sampling methods such as umbrella sampling [23], steered MD [24], metadynamics [25], adaptive-biasing force [26], and accelerated MD [27]. Such methods are often used in estimating the free energy change in two-state problems like binding of a ligand or closure of a gate. For example, the potential of mean force (PMF) for binding of a ligand can be calculated from umbrella sampling MD simulations using the weighted histogram analysis method [28], or from steered MD simulations using Jarzynski’s equation [29].

### 2.2. Free Energy Calculations

As stressed above, most biological processes are not amenable to brute-force MD simulations. This is certainly the case for the glutamate transporters—the full transport cycle takes about three min in Glt${}_{\mathrm{Ph}}$ and 10 ms in EAAT3 [21], which are far beyond the simulation capabilities of current supercomputers. In such situations, free energy calculations with enhanced sampling techniques can be performed to study the mechanism and energetics of the transport process, e.g., they can provide information on binding free energies of ligands, gating motion, and conformational changes of proteins. Because results of free energy calculations can be directly compared to experiments, they play an important role in validation of MD simulations. Free energy calculations can be classified into two groups according to whether they are path-independent or path-dependent, which are discussed in more detail below.

In the path-independent class, a group or all atoms of a molecule are alchemically transformed to another group or void. For example, in binding free energy calculations, a ligand is progressively annihilated in its binding site while it is created in bulk. This alchemical transformation determines the free energy required to translocate the ligand from the binding site to the bulk and is called the interaction free energy, $\Delta G_{\text{i}nt}$. There are two main methods for calculating $\Delta G_{\text{i}nt}$ through an alchemical transformation: free energy perturbation (FEP) and thermodynamic integration (TI). In both methods, a hybrid Hamiltonian is introduced in the form of

(1) $$H\left(\lambda \right)=\left(1-\lambda \right)H_{0}+\left(\lambda \right)H_{1}$$

Here, $H_{0}$ and $H_{1}$ represent the initial and final states, which could be the ligand in the binding site and in the bulk, respectively. As the coupling parameter *λ* is varied from 0 to 1, the ligand is transformed from the binding site to the bulk. In the FEP method, the coupling parameter is divided into *n* subintervals, and the change in free energy for each window is calculated from the ensemble average

(2) $$\Delta G_{i}=-kT\ln{\langle \exp\left[-\left(H\left(\lambda_{i+1}\right)-H\left(\lambda_{i}\right)\right)/kT\right]\rangle }_{\lambda_{i}}$$

where *k* is the Boltzmann constant and *T* is the temperature.

The free energy difference for the whole transformation is given by the sum, $\Delta G_{\text{i}nt}=\sum_{i}\Delta G_{i}$. Here, the subintervals are chosen such that $\Delta G_{i}$ in each window remains around a few kcal/mol, otherwise sampling problems are likely to occur.

In the TI method, the free energy difference is calculated from the integral

(3) $$\Delta G_{\text{i}nt}=\int_{0}^{1}{\langle \frac{\partial H(\lambda )}{\partial \lambda }\rangle }_{\lambda }d\lambda$$

Here, the ensemble average of the derivative of the Hamiltonian with respect to *λ* is determined from MD simulations at several *λ* values, and the results are numerically integrated. Using Gaussian quadrature, this integral can be evaluated with a small number of windows. Thus, TI can be advantageous over FEP because it permits longer sampling of smaller number of windows. For both methods, it is important to calculate both the forward and backward transformations to check for hysteresis effects. Traditionally, alchemical transformations for ligands are performed separately for the annihilation in the binding site and creation in the bulk. It has been known that this could lead to substantial errors especially for charged molecules. An obvious reason is that the system must be kept neutral in MD simulations, but also errors arising from simulation artifacts will not necessarily cancel when the systems used in the binding site and bulk calculations are very different. This problem can be overcome by performing the alchemical transformations in the binding site and bulk in the same system and simultaneously as shown in ligand binding studies to glutamate receptor [30].

The quantity measured in ligand binding experiments is the binding constant, $K_{\text{e}q}$, which is related to the standard binding free energy by

(4) $$\Delta G_{b}=-kT\ln\left(K_{eq}C_{0}\right)$$

where $C_{0}=1/(1661$ Å${}^{3})$ is the standard concentration of 1 M. In the path-independent methods, the standard binding free energy of a ligand is given by [31]

(5) $$\Delta G_{\text{b}}=\Delta G_{\text{int}}+\Delta G_{\text{tr}}+\Delta G_{\text{rot}}+\Delta G_{\text{con}}$$

Here, the first term on the right hand side is the interaction free energy determined from FEP/TI calculations as discussed above. The second and third terms account for the translational and rotational entropy loss upon binding, which are determined from the fluctuations of the ligand in the binding site. The last term accounts for the entropy loss arising from application of conformational restraints. The last two terms are required for molecules which can rotate and have internal degrees of freedoms, but not for ions which are treated as point particles.

Alchemical transformation methods work fine for small molecules which are neutral or lightly charged. For large or highly charged molecules, these methods lose their reliability due to substantial errors in calculation of hydration energies. The alternative is to use the path-dependent PMF methods, where the free energy profile of a ligand is calculated from the binding site to the bulk. The binding constant is determined from the 3D integral of the PMF, W(r)

(6) $$K_{eq}=\int_{site}e^{-W\left(r\right)/k_{B}T}\;d^{3}r$$

Because calculation of the PMF in a 3D grid is computationally prohibitive, a 1D approximation is usually invoked, and the PMF is determined along a reaction coordinate only. Assuming a flat-bottomed potential in transverse directions, the integral in Equation (6) can be evaluated along the reaction coordinate *ξ* as

(7) $$K_{eq}=\pi R^{2}\int_{\xi_{1}}^{\xi_{2}}e^{-W(\xi )/kT}\;d\xi$$

where $\xi_{1}$ and $\xi_{2}$ refer to the ligand’s center of mass (COM) coordinates in the binding site and bulk, respectively. The factor $\pi R^{2}$ measures the average cross-sectional area of the binding pocket and is determined from the transverse fluctuations of the COM of the ligand [32]. The binding free energy follows from Equation (4) as before.

The standard method for PMF calculations is umbrella sampling MD simulations, which is an equilibrium method. Here, biasing harmonic potentials are employed at a number of windows along the reaction coordinate to enable sampling of the ligand at high-energy points. The COM coordinates of the ligand collected during the umbrella sampling simulations are unbiased and the PMFs obtained from the Boltzmann equation at each window are combined using the weighted histogram analysis method [28]. An alternative non-equilibrium method for PMF calculations is to determine the work done on the ligand while it is pulled from the binding site to bulk in a number of steered MD simulations and perform a Boltzmann average of the work functions using Jarzynski’s equation [29]. While this method is much simpler to implement, it is not as accurate as umbrella sampling [33].

The choice of the reaction coordinate is clearly very important in PMF calculations. In cases such as ion channels, the channel axis provides a natural choice for the reaction coordinate which can be easily implemented. However, when the binding site is buried inside a protein—as it happens in glutamate transporters and receptors—there is usually no straight path connecting the binding site to bulk. In such cases, the reaction coordinate follows a curved trajectory, complicating the PMF calculations. Use of the path-independent methods may be more advantageous in such situations, provided they are feasible and accurate [30].

## 3. MD Simulations of Glutamate Transporters

### 3.1. Prokaryotic Homolog Glt${}_{\mathrm{Ph}}$

**Figure 2:**
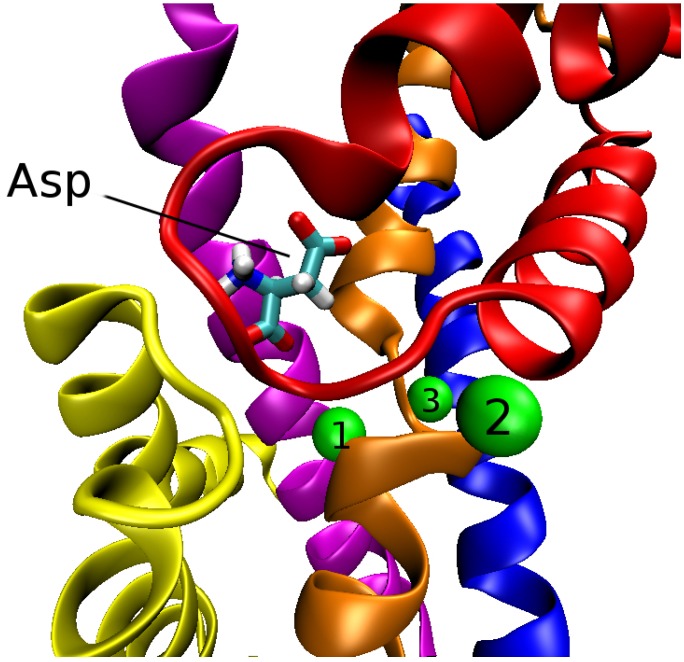
Transport domain of a subunit of Glt${}_{\mathrm{Ph}}$ depicting the positions of Asp and three Na${}^{+}$ ions (green spheres numbered from 1 to 3). The TM segments and hairpins involved in the coordination of Asp and Na${}^{+}$ ions are indicated as follows: HP1 (**yellow**); HP2 (**red**); TM3 (**blue**); TM7 (**orange**) and TM8 (**magenta**). Upward motion of HP2 opens the outward gate and exposes the ligands to water.

Glt${}_{\mathrm{Ph}}$ exists as a trimer in the membrane with three identical subunits held together by non-covalent bonds. Within each subunit (or monomer) there are eight transmembrane segments (TM1–TM8) and two hairpin loops (HP1 and HP2) between TM6 and TM8. In the outward state, the trimer has a bowl shaped structure with a diameter of about 50 Å and depth 30 Å. The trimer can be grouped into two domains: trimerization and transport. The trimerization domain consists of TM1, 2, 4 and 5 from each subunit, whose function is to stabilize the trimer complex. The transport domain consists of TM3, 6, 7 and 8 along with the two hairpins, HP1 and HP2 from each subunit. The structure of the transport domain of Glt${}_{\mathrm{Ph}}$ with the bound aspartate and three Na${}^{+}$ ions is shown in Figure 2.

The crystal structure of Glt${}_{\mathrm{Ph}}$ resolved in 2007 identified the binding sites for the substrate and two Na${}^{+}$ ions labelled Na1 and Na2 [17]. As the stoichiometry of ion coupling was not known at that time, initial MD simulations of Glt${}_{\mathrm{Ph}}$ were performed using this structure [34,35], where opening of the HP2 gate was demonstrated to be the outward gate. Subsequent radio-labelled experiments with ${}^{22}$Na${}^{+}$ ions indicated that three Na${}^{+}$ ions are co-transported in Glt${}_{\mathrm{Ph}}$ [36]. Several binding sites were proposed for the third Na${}^{+}$ ion (labelled Na3) from computational results and experimental observations [37,38,39,40,41]. According to the mutagenesis experiments on EAAT3 [39,42], the side chains of residues T92 and D312 are involved in the coordination of one of the co-transported Na${}^{+}$ ions during transport. Because neither residues coordinate Na1 or Na2 in the crystal structure of Glt${}_{\mathrm{Ph}}$ [17], the side chains of these residues indicate a possible binding site for Na3. This argument was strengthened by the observation that in MD simulations of Glt${}_{\mathrm{Ph}}$ with Na1 and Na2 ions, the D312 side chain was observed to flip and start coordinating Na1 [38,41]. The resulting coordination shell for Na1 is in conflict with the crystal structure of Glt${}_{\mathrm{Ph}}$, underscoring the need for a cation at the site of D312 side chain to prevent its flipping. Such an Na3 binding site was first proposed from electrostatic calculations, consisting of the side chains of Y88, T92, N310 and D312, and the backbone of G404 [39]. Refinement of this site in MD simulations using the closed structure of Glt${}_{\mathrm{Ph}}$ resulted in a slightly different Na3 site, with the side chains of T92, N310 and D312 retained but Y88 and G404 replaced by a water molecule [40]. Yet a third Na3 site was proposed from the MD simulations of TBOA-bound open structure of Glt${}_{\mathrm{Ph}}$, where the side chains of T92, N310 and D312 were again retained but the coordination shell was completed with the side chain of S93 and the backbone of Y89. The difference between the two MD simulations is caused by the flipping of the N310 side chain between the closed and open structures of Glt${}_{\mathrm{Ph}}$. Mutagenesis experiments (in particular, S93A [41]) indicated that the last proposed site was the most likely site for binding of Na3.

With the binding site for Na3 established, the Glt${}_{\mathrm{Ph}}$ model was ripe for free energy calculations for binding of substrate and Na${}^{+}$ ions. Such calculations were performed first for the outward-facing conformation of Glt${}_{\mathrm{Ph}}$ [43], followed by calculations for the inward-facing conformation [44]. The results summarized in Table 1 show that

(i) the order of binding of the ligands in the outward-facing conformation is Na3, Na1, Asp, (Outward gate closes), and Na2 (the Na2 site forms only after HP2 gate closes);
(ii) the binding free energies of the ligands are very similar in the outward- and inward-facing conformations, consistent with the observation that the binding pockets are preserved in the corresponding crystal structures;
(iii) release of the ligands in the inward-facing conformation follows the reverse of the order of binding, that is, Na2, (Inward gate opens), Asp, Na1, and, Na3.

**Table 1:** Ligand binding free energies, $\Delta G_{b}$, of Na${}^{+}$ and Asp in Glt${}_{\mathrm{Ph}}$ for both outward- and inward-facing conformations (units, kcal/mol). The ligands present during the free energy calculations are indicated in parenthesis (other combinations of ligands yield higher free energies, and therefore are not shown) [43,44].

Ligand	Outward	Inward
Na3	–18.7 ± 1.1	–16.3 ± 1.1
Na1 (Na3)	–7.1 ± 1.3	–7.3 ± 1.3
Asp (Na1, Na3)	–3.8 ± 1.0	–4.9 ± 1.1
Na2 (Na1, Na3, Asp)	–2.7 ± 1.3	–2.4 ± 1.2

We note that the Na2 binding site used in the free energy calculations is slightly shifted from its position observed in the crystal structure [17]. This could be due to the substitution of Tl${}^{+}$ for Na${}^{+}$ in experiments. Unfortunately, probing the Na2 site with mutagenesis experiments is very difficult because the coordinating atoms are mostly backbone carbonyl oxygens. Na2 is bound to TM7 and HP2 carbonyl oxygens, and is exposed to water. Thus the binding site is formed only after the HP2 gate closes, and binding of Na2 is proposed to lock this gate [17]. Presence of Na2 is expected to prevent water molecules disrupting the hydrogen bonds between HP1 and HP2 and thereby open the gate. While the calculated Na2 binding free energy is quite small, conformational changes occurring after the gate closure may lead to a more stable Na2 binding site [45]. The effect of the substrate transport on the Na2 site was also investigated in a recent steered MD study [46]. The simulation results indicated maturing of the Na2 site proposed earlier [43] during the substrate transport, which was validated in mutation experiments [46].

**Figure 3:**
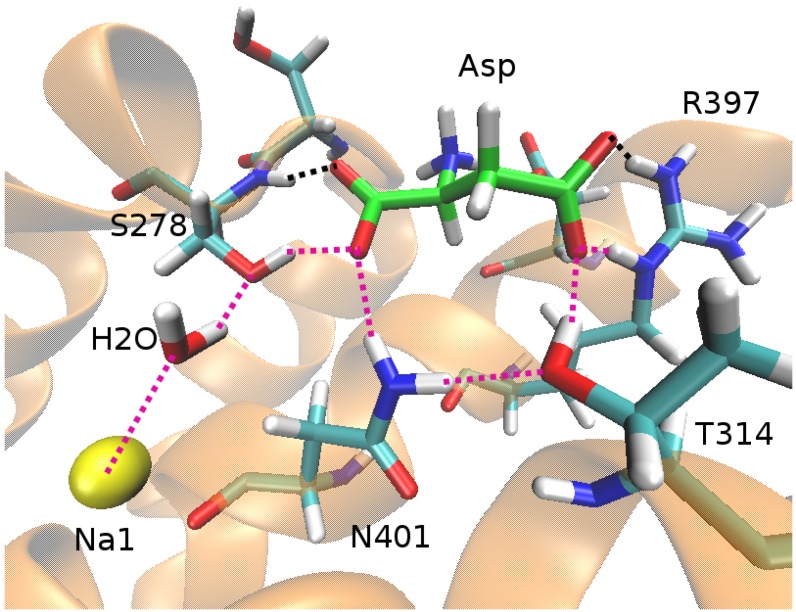
The binding pocket of Glt${}_{\mathrm{Ph}}$ in the open state of the outward-facing conformation, showing the hydrogen-bond network (**purple dotted lines**) that couples Na1 (**yellow sphere**) to Asp (**green backbones**).

An important issue clarified by the free energy calculations is the coupling between the substrate and the Na${}^{+}$ ions. In some secondary transporters (e.g., LeuT), a Na${}^{+}$ ion is in direct contact with the substrate so the coupling mechanism is obvious and the strength is assured. In Glt${}_{\mathrm{Ph}}$, however, the closest Na${}^{+}$ ion to Asp is Na1, which is 7 Å away from the nearest carbonyl oxygen of Asp, separated by a water molecule and the side chain of S278 (Figure 3). Nevertheless, the hydrogen-bond network induced by Na1 appears to be sufficient to stabilize Asp in the binding site. Conversely, removal of Na1 leads to the disruption of the hydrogen-bond network and Asp is released to the solvent in a few ns [43]. Because of the fast release of Asp in the absence of Na1, it was not possible to estimate the Asp–Na1 coupling free energy in the outward-facing conformation. A more stable Asp in the inward-facing conformation allowed such a calculation, and the presence of Na1 was found to change the binding free energy of Asp from +1.8 kcal/mol to −4.9 kcal/mol [44]. Thus the presence of Na1 is essential for binding of Asp with the Asp–Na1 coupling contributing −6.7 kcal/mol to its binding free energy.

It is important to stress that the results in Table 1 are based on the crystal structures and do not take into account any conformational changes that may occur during the ligand binding or release processes [47]. For example, the path to Na3 goes through the Na1 site and is blocked by a hydrogen-bond network in the apo state [40]. Thus, binding of the first two Na${}^{+}$ ions are expected involve some conformational change and may even be coupled to facilitate the breaking of the hydrogen-bond network. In the case of ligand release, however, there is less scope for such a coupling, and the very low binding free energy predicted for Na3 is likely to stand. Then unbinding of Na3 is expected to be the rate-limiting step in the transport cycle. This is supported by the mutation of the residues coordinating Na3, which reduces its binding affinity and thereby increase the transport rate by 20-fold. Estimates for the release time of Na3 obtained from Kramer’s rate theory give consistent results with the experimental turn over rates in Glt${}_{\mathrm{Ph}}$, providing further support for this hypothesis [44].

Conformational changes that occur in Glt${}_{\mathrm{Ph}}$ during the transport cycle can be divided into three groups: (i) gating motions and ligand binding in the outward-facing state; (ii) transition of the transport domain from the outward-facing to the inward-facing state; (iii) gating motions and ligand release in the inward-facing state. The gating motions and substrate binding/release in (i) and (iii) are relatively fast and localized events while the transition in (ii) is slow and highly non-local. Therefore, the former have been studied using MD and metadynamics simulations but the latter had to be studied using coarse-grained methods such as anisotropic network model (ANM) [48].

The unique role of HP2 in opening of the outward gate was well established from the early MD simulations [34,35]. From symmetry arguments, HP1 was initially proposed as the inward gate [18,49], and this was partially supported from metadynamics simulations where a larger movement of HP1 compared to HP2 was predicted [50]. However, the opposite has been observed in unbiased MD simulations of the inward-facing state, namely, HP1 moves little and preserves its contacts with the substrate while HP2 moves further and loses some contacts with the substrate [44,51,52]. In particular, the multiple microsecond MD simulations performed in the last work [52] has clearly established the dominant role of HP2 as the inward gate. The relatively smaller opening of HP2 in the inward gate compared to the outward gate happens because HP2 is partially buried inside the protein in the former case and thus has less scope to move. In metadynamics [50] and long MD [52] simulations, the release of Na2, Asp, and Na1 to cytoplasm was also observed, and the order of release was consistent with that predicted from free energy calculations. Unfortunately, Na3 was not included in these simulations. Clarification of the role of Na3 in limiting the rate of transport will be of great interest through independent computational studies.

Computational investigation of the outward → inward transition of the transport domain using MD simulations is much more demanding, and has been rarely attempted. For example, a steered MD simulation of this transition did not yield satisfactory results presumably due to insufficient sampling [53]. Instead progress has been made using ANMs in combination with MD simulations [54,55,56,57]. These studies have shown that the transition occurs independently in each subunit involving large scale collective motion of the binding pocket [54], and the transport and trimerization domains move in opposite directions along the membrane normal [55]. Use of ANMs to study large scale domain motions was justified through comparison of the results to long MD simulations [56]. Finally, the transition pathway of the transport domain between the outward and inward states was constructed using a two-state ANM [57]. Complementary experimental work has been done using single molecule FRET imaging and indicate that each subunit in the trimer moves independent of the others and the transition is stochastic [58,59,60].

A common feature of the glutamate transporter family is the existence of a Cl${}^{-}$ channel within the transporter [21]. The Cl${}^{-}$ channel is conserved among glutamate transporters, indicating its importance in functioning of the transporter. The Cl${}^{-}$ channel is activated by the binding of the substrate and Na${}^{+}$ ions but is uncoupled from the transport cycle as demonstrated by the mutation of residues in EAATs, which block the substrate transport but does not impede Cl${}^{-}$ conductance [61]. So far, there are no crystal structures of Glt${}_{\mathrm{Ph}}$ with an open Cl${}^{-}$ channel, which has impeded computational study of Cl${}^{-}$ conductance. Very recently, a model of Glt${}_{\mathrm{Ph}}$ with an open Cl${}^{-}$ channel has been constructed [62], using the experimental observation that the channel is partly formed by an aqueous cavity at the interface of the transport and trimerization domains [63]. In this study [62], a membrane potential of ±1.6 V was applied to both the outward- and inward-facing states of Glt${}_{\mathrm{Ph}}$ in order to create such a cavity, but an open channel conformation was not found during eight μs MD simulations. This suggested that the Cl${}^{-}$ channel may be formed in an intermediate state of the transporter during substrate translocation. Intermediate states of Glt${}_{\mathrm{Ph}}$ were then generated using essential dynamics sampling—a rare event sampling method. Application of a membrane potential in an intermediate state indeed resulted in separation of the trimerization and transport domains, creating a water filled open channel conformation. The proposed model [62] is mostly consistent with experimental observations, e.g., the predicted pore diameter is 5.6 Å while the experimental values are in the range 5–6 Å [21], and the R276S mutation at the centre of the pore converts it to a Na${}^{+}$ channel as observed experimentally [64].

### 3.2. Excitatory Amino Acid Transporters

There are no crystal structures for EAATs as yet, so homology models based on the Glt${}_{\mathrm{Ph}}$ structure need to be constructed for computational investigation of EAATs. While the overall sequence identity between Glt${}_{\mathrm{Ph}}$ and EAATs is low, it is over 60% in the binding pocket. Thus homology models may be able to explain the functional differences between Glt${}_{\mathrm{Ph}}$ and EAATs, namely, co-transport of H${}^{+}$ and counter-transport of K${}^{+}$ ion. To highlight the functional similarity between Glt${}_{\mathrm{Ph}}$ and EAATs, we compare in Table 2 all the important residues in the binding pocket identified from the crystal structures and mutation experiments. Focusing on EAAT3 as there are more data on it, we see that only five residues differ from Glt${}_{\mathrm{Ph}}$. Of these, R276 and T352 in Glt${}_{\mathrm{Ph}}$ contribute backbone carbonyls to the coordination of the substrate and Na2, respectively, and the other three are not involved in the coordination of the ligands. Thus we expect the coordination shells of the Na${}^{+}$ ions and the substrate to be preserved. Moreover, the mutations, R276 → S331 and M395 → R445, transfer the position of arginine from HP1 in Glt${}_{\mathrm{Ph}}$ to TM8 in EAAT3 but do not change the location of its side chain. Thus, they preserve the structural similarity. The only other significant mutation in Table 2 is Q318 → E374. The E374 residue has been proposed to be the protonation site in EAAT3 from mutation experiments—the E374Q mutation does not affect the Glu affinity but abolishes the pH dependence of Glu transport [65,66].

**Table 2:** Glt${}_{\mathrm{Ph}}$ residues involved in the coordination of the ligands and their equivalents in excitatory amino acid transporters EAAT1, EAAT2 and EAAT3 (human sequences are used). Residues from the mutagenesis experiments are also included in the table. The residues that are not conserved between Glt${}_{\mathrm{Ph}}$ and EAATs are indicated with red.

Glt${}_{\mathrm{Ph}}$	Y89	T92	S93	Q242	R276	S277	S278	G306	T308
EAAT1	Y127	T130	T131	H328	S363	S364	S365	G394	T396
EAAT2	Y124	T127	T128	H326	A361	S362	S363	G392	T394
EAAT3	Y98	T101	T102	H296	S331	S332	S333	G362	T364
Glt${}_{\mathrm{Ph}}$	N310	D312	T314	Y317	Q318	S349	I350	T352	G354
EAAT1	N398	D400	T402	Y405	E406	S437	I438	A440	G442
EAAT2	N396	D398	T400	Y403	E404	S435	I436	A438	S440
EAAT3	N366	D368	T370	Y373	E374	S405	I406	A408	G410
Glt${}_{\mathrm{Ph}}$	V355	G359	D390	D394	M395	R397	T398	N401	D405
EAAT1	I443	G447	D472	D476	R477	R479	T480	N483	D487
EAAT2	I441	G445	D470	D474	R475	R477	T478	N481	D485
EAAT3	V411	G415	D440	D444	R445	R447	T448	N451	D455

Homology models for EAAT3 in the outward- and inward-facing states have been recently constructed using the available crystal structures of Glt${}_{\mathrm{Ph}}$ [67]. MD simulations of these EAAT3 models have revealed that:

(i) Glu is stably bound when E374 is protonated but becomes unstable when E374 is deprotonated in both the outward and inward-facing states. Thus binding of Glu is contingent upon the protonation of E374, which is consistent with the experimental observations indicating E374 as the protonation site [65,66].
(ii) The coordination shells for Na1, Na3, and Glu are very similar to those in Glt${}_{\mathrm{Ph}}$, consistent with the expectations from the alignment diagram in Table 2. The Na2 coordination is somewhat different from that of Glt${}_{\mathrm{Ph}}$ in that S405 carbonyl and T364 hydroxyl are not involved in the coordination of Na2, and Na2 is not stably bound. It is possible the Na2 site is not conserved in EAATs. Further experimental and computational work is required to determine the Na2 binding site in EAATs.
(iii) Gating in the outward-facing state is very similar to that in Glt${}_{\mathrm{Ph}}$ but a rather different mechanism occurs in the inward-facing state—HP1 and HP2 move about similar amounts, leading to a much larger opening of the gate compared to that in Glt${}_{\mathrm{Ph}}$. This can be traced to the transfer of an arginine (R276) from HP1 in Glt${}_{\mathrm{Ph}}$ to TM8 in EAAT3 as mentioned above. In Glt${}_{\mathrm{Ph}}$, R276 forms a salt bridge with D394, which prevents opening of HP1. Transfer of this arginine (R445) to TM8—which still makes a salt bridge with D444—enables larger opening of HP1, facilitating the release of the larger Glu substrate.
(iv) A number of sites have been proposed for binding of a K${}^{+}$ ion in the inward-facing state of EAATs. The most likely K${}^{+}$ sites, together with the complete coordination shells obtained from the MD simulations of the EAAT3 model, are listed in Table 3. Site 1 is very similar to the Na1 site, and is proposed because the mutations that turn the transporter into an exchanger (e.g., D455N) also impair its interaction with a K${}^{+}$ ion [68,69]. Site 2 corresponds to the proton binding site—when the proton leaves, K${}^{+}$ could bind there to neutralize the site [70]. The last site overlaps with the substrate *α*-amino group, and was predicted from electrostatic mapping calculations [37]. To assess the likelihood of each site being the K${}^{+}$ site, binding free energies and the K${}^{+}$/Na${}^{+}$ selectivity free energies were calculated (Table 3). The selectivity free energies were calculated to check the hypothesis that the last Na${}^{+}$ ion is exchanged with a K${}^{+}$ ion in EAATs in order to speed up the very low transport rates observed in Glt${}_{\mathrm{Ph}}$ [67]. Site 1 appears to be the most likely K${}^{+}$ site as it has the largest affinity for K${}^{+}$. Also it is consistent with the K${}^{+}$–Na${}^{+}$ exchange hypothesis as it has negligible K${}^{+}$/Na${}^{+}$ selectivity. Recent crystal structure of Glt${}_{\mathrm{Tk}}$—a close homolog of Glt${}_{\mathrm{Ph}}$ resolved in the apo state—provides further experimental support for this site [71]. The Glt${}_{\mathrm{Tk}}$ structure exhibits some conformational differences from that of Glt${}_{\mathrm{Ph}}$ in the vicinity of site 1, which are well reproduced in the computational model.

**Table 3:** EAAT3 residues coordinating the K${}^{+}$ ion at three proposed binding sites, and their respective standard binding free energies and K${}^{+}$/Na${}^{+}$ selectivity free energies (in units of kcal/mol).

	Site 1	Site 2	Site 3
Helix-Residue	TM7–G362 (O)	TM7–T370 (OH)	HP1–S331 (O)
	TM7–I365 (O)	TM7–T370 (O)	HP1–S331 (OH)
	TM7–N366 (O)	TM7–E374 (O${}_{1}$)	TM8–D444 (O)
	TM8–D455 (O${}_{1}$)	TM7–E374 (O${}_{2}$)	TM8–D444 (O${}_{1}$)
	TM8–D455 (O${}_{2}$)	H2O (1)	TM8–D444 (O${}_{2}$)
	H2O (1)	H2O (2)	TM8–T448 (OH)
	H2O (2)		H2O
$\Delta G_{b}$ (K${}^{+}$)	–20.5 ± 1.1	–9.5 ± 1.2	–6.5 ± 0.8
$\Delta G_{sel}$ (K${}^{+}$/Na${}^{+}$)	0.5 ± 0.4	3.9 ± 0.4	–3.1 ± 0.4

The EAAT3 model discussed above [67] confirms that the Glt${}_{\mathrm{Ph}}$ structure provides a working model for functional studies of EAAT3. Furthermore, it provides a rationale for K${}^{+}$ counter-transport through the K${}^{+}$–Na${}^{+}$ exchange mechanism, which speeds up the transport cycle in EAATs compared to Glt${}_{\mathrm{Ph}}$. It also explains the need for H${}^{+}$ co-transport to keep the charge content the same as in Glt${}_{\mathrm{Ph}}$, that is, the fully bound EAAT3 with protonated E374 in the outward-facing state and the K${}^{+}$ bound EAAT3 with deprotonated E374 in the inward-facing state have exactly the same charge content as the corresponding states in Glt${}_{\mathrm{Ph}}$. Thus, the energetic cost of the ion-coupled substrate transport across the membrane in EAAT3 remains the same as in Glt${}_{\mathrm{Ph}}$.

In a subsequent paper, this EAAT3 model was used in a detailed study of the proton transport mechanism through p*K*${}_{a}$ calculations [72,73]. p*K*${}_{a}$ of all the acidic residues in the transport domain were calculated in the fully-bound outward-facing state of EAAT3. E374 was found to be the only acidic residue with a high enough p*K*${}_{a}$ value to be protonated, confirming it as the protonation site. p*K*${}_{a}$ values of E374 in different states of EAAT3 were then calculated to get a better understanding of the proton transport mechanism. In the outward-facing state, the p*K*${}_{a}$ value of E374 was 11.7 in the apo state, went down to 6.6 after the binding of Na1 and Na3, went up to 10.4 after Glu binding, and to 19.1 after the gate closure. These p*K*${}_{a}$ values, in combination with the fact that Glu binds after Na1 and Na3, and Glu is not stable with a deprotonated E374, suggest that proton and Glu binding are mutually coupled and occur simultaneously. The closure of the gate completely isolates E374 from solvent, leading to the dramatic rise in the p*K*${}_{a}$ value. Similar p*K*${}_{a}$ values of E374 were obtained in the inward-facing state, which meant that the E374 would not be deprotonated in the apo state. Placement of a K${}^{+}$ ion in the three proposed sites (Table 3) with a protonated E374 suggested a possible proton release mechanism. The p*K*${}_{a}$ values with a K${}^{+}$ placed in sites 1 and 3 were reduced from 11.7 in the apo state to 4.7 and 6.9, respectively, and the K${}^{+}$ ion in site 2 was stable only with a deprotonated E374. These observations indicate that binding of a K${}^{+}$ ion is required for the deprotonation of E374 and completion of the transport cycle. Thus, in the most likely scenario, the proton and Glu are released simultaneously, and the exchange of the last Na${}^{+}$ ion with K${}^{+}$ prevents protonation of E374.

### 3.3. Neutral Amino-Acid Transporters

ASCTs transport small neutral amino acid across the plasma membrane and belong to the same solute carrier family as Glt${}_{\mathrm{Ph}}$ and EAATs. ASCT1 is selective to alanine, serine and cysteine as the acronym suggests, however, it can also transport threonine, asparagine and proline. ASCT2, on the other hand, have a broader selectivity profile and plays an important role in glutamine regulation. Very little computational work has been done on ASCTs because there are no crystal structures and relatively smaller number of mutation studies are available compared to EAATs to guide modeling efforts. Originally, coupling of a single Na${}^{+}$ ion to the substrate was proposed for the transport cycle [74,75]. However, coupling of three Na${}^{+}$ ions to Glu/Asp in EAATs and Glt${}_{\mathrm{Ph}}$ suggests that two Na${}^{+}$ ions may be required for an efficient transport of neutral amino acids. Similarity of the sequences of ASCTs with Glt${}_{\mathrm{Ph}}$ in the transport domain, and the preservation of the the three Na${}^{+}$ binding sites found in Glt${}_{\mathrm{Ph}}$ provide further support for this argument. In a recent study, the Na${}^{+}$ dependence of the anion current in ASCT2 was found to be biphasic, which suggests binding of at least two Na${}^{+}$ ions to ASCT2 [76]. In this work, MD simulations were also performed on a homology model of ASCT2—three Na${}^{+}$ ions and Ala placed at the ligand binding sites as found in Glt${}_{\mathrm{Ph}}$ remained stably bound for 8 ns. Unfortunately, these MD simulations are too short for an adequate sampling of the phase space, and need to be extended and supported by free energy calculations to determine the stoichiometry of the Na${}^{+}$ coupling in ASCTs.

In a more recent study, the Na${}^{+}$ sites in ASCT1 were investigated using both mutagenesis experiments and MD simulations [77]. Mutation of the aspartate residues coordinating the Na${}^{+}$ ions at either the Na1 or the Na3 site to alanine affected the Na${}^{+}$ affinity and diminished the substrate exchange but did not stop it. This suggests that binding of a Na${}^{+}$ ion at either site is sufficient for the transport of the substrate albeit at much reduced rates. In MD simulations of the homology model of ASCT1, all three Na${}^{+}$ sites from Glt${}_{\mathrm{Ph}}$ were included in their respective sites in ASCT1, and Asp was replaced with Ser. The system was found to be stable during 20 ns of simulations. This is again too short to conclude that ASCT1 can stably bind three Na${}^{+}$ ions. Further MD simulations of ASCT1 and its mutated forms were performed for various configurations of ligands and mutations—including an additional Na1${}^{\prime }$ site, which corresponds to the site obtained in MD simulations of Glt${}_{\mathrm{Ph}}$ when Na3 was not included [41]. The results obtained from 12 MD simulations were mostly consistent with the mutation experiments.

## 4. Conclusions and Future Prospects

Determination of the crystal structures of Glt${}_{\mathrm{Ph}}$—a prokaryotic homolog of glutamate transporters—ushered a new age in studies of glutamate transporters where the focus is on a molecular-level understanding of the transport process. Computational methods will play a critical role in this effort as many steps in the transport process are not directly accessible by experiments. Already many computational studies of Glt${}_{\mathrm{Ph}}$ have been performed, leading to a better understanding of the transport mechanism. There are still some open questions on Glt${}_{\mathrm{Ph}}$, for example, binding and release of Na${}^{+}$ ions, whose resolution will require a more subtle approach than brute force MD simulations.

The real value of the Glt${}_{\mathrm{Ph}}$ structures are, of course, in that they provide templates for constructing homology models for the mammalian glutamate and other amino acid transporters, so that their transport mechanism can be studied using computational methods. Progress on these fronts have been slower with only a few papers published so far. Nevertheless, the results of these papers are very encouraging, and it is hoped that some of the outstanding questions on EAATs and ASCTs will be resolved in the near future through a combined approach involving computational prediction and experimental verification.
